# Coping Strategies in Patients with Acquired Brain Injury: A Scoping Review

**DOI:** 10.3390/brainsci14080784

**Published:** 2024-08-01

**Authors:** Davide Cardile, Andrea Calderone, Maria Pagano, Irene Cappadona, Carmela Rifici, Angelo Quartarone, Francesco Corallo, Rocco Salvatore Calabrò

**Affiliations:** IRCCS Centro Neurolesi Bonino-Pulejo, S.S. 113 Via Palermo, C.da Casazza, 98124 Messina, Italy

**Keywords:** acquired brain injuries, traumatic brain injuries, neurorehabilitation, coping, functional recovery

## Abstract

In recent years, there has been marked interest in looking at the psychological consequences of medical conditions, such as traumatic or acquired brain injuries. Coping strategies are essential for clinical recovery and for dealing with the stressful events that a clinical condition brings with it. The purpose of this review is to analyze studies that explore how coping strategies influence psychological changes in patients with acquired brain injury. Studies were identified from research in the PubMed, Scopus, and Embase databases. According to our findings, patients with ABI utilize different coping strategies based on the circumstances and factors such as the diagnosis severity, their age, time lived with the pathology, and personal characteristics, which have an influence on quality of life and rehabilitation. This review demonstrated that coping strategies have an impact on different aspects of the clinical and personal lives of patients with ABI. The rehabilitation process must consider the influence of these mechanisms on dealing with situations, as they can change cognitive and emotional perceptions of patients’ experience with the disease, as well as laying the foundations for functional or dysfunction in terms of the propensity of a person for the path of psychological and physical recovery.

## 1. Introduction

In recent years, psychologists have become increasingly interested in exploring the psychological effects of medical conditions. One prevalent neurological disorder is acquired brain injury (ABI), which refers to brain damage occurring after birth on the basis of either traumatic or non-traumatic events, such as ischemic or hemorrhagic stroke [1]. This clinical condition is currently the leading cause of death and disability among young adults and is regarded as one of the most prevalent neurological disorders [1].

An epidemiological study of clinical databases states that the estimated global incidence of ABI is 1 per 500 people, with the highest occurrence rates in children under 4, adults under 30, and individuals over 65 years old [2]. A recent study on the Italian population between 2012 and 2021 estimates an ABI rate of 77.30 per 100,000 people, higher among males, with exponential growth after the age of 70. The main causes are domestic accidents (33.1 percent), with the elderly and children predominating, and road accidents (17.7 percent), which mainly affect males aged 15–24 and the elderly [3]. This demographic profile indicates that there are a significant number of ABI survivors worldwide with a substantial life expectancy. Acquired brain injury results in a considerable burden of physical, cognitive, and psychosocial consequences for survivors, and it can also elevate the risk of developing neurodegenerative diseases later in life [4]. Research has shown that cognitive and behavioral changes can occur in almost all forms of ABI. However, brain injury is a highly complex phenomenon with widely varied effects, meaning that no two individuals will experience the same outcomes or challenges [1]. Since the brain governs every aspect of human life—physical, behavioral, social, intellectual, and emotional—damage to it will inevitably impact some area of a person’s life [5]. The outcome of an injury largely depends on its nature and severity, and appropriate treatment is crucial for determining the level of recovery [4]. Individuals who suffer from an acquired brain injury must undergo a challenging process of psychological adaptation, which can be understood in terms of coping behavior. Coping is defined by Lazarus and Folkman (1984) as the constantly evolving cognitive and behavioral efforts to manage specific external and internal demands that are perceived as taxing or exceeding the resources of the individual. Their stress and adaptation theory has significantly shaped the coping research field [6]. This theory suggests that an individual defines a situation as stressful based on their unique appraisal of the event rather than the event’s objective characteristics. Emotional distress occurs when an individual perceives their environmental demands as surpassing their resources. The term “coping” is used irrespective of whether the process results in adaptive or maladaptive outcomes. Once an event is appraised as stressful, coping strategies are employed and are generally classified as either problem-focused or emotion-focused [7].

Adaptive functioning encompasses proficiency in three key areas as defined by the American Association on Intellectual and Developmental Disabilities (AAIDD): conceptual skills (such as language and literacy), practical skills (like self-care and safety precautions), and social abilities (such as interpersonal skills and problem-solving in social situations) [8].

Many individuals with acquired brain injury struggle with processing multiple stimuli at once, leading to difficulties in focusing attention, filtering out irrelevant information, and shifting attention quickly [1]. These impairments can result in cognitive sluggishness and overwhelm, leading to mental fatigue and increased stress. As a result, these individuals may have a reduced ability to cope with stressors that are beyond their control. Coping mechanisms may involve problem-solving and emotional regulation and can vary depending on the specific task, situational demands, and the individual’s abilities. Awareness of one’s own capabilities, limitations, and motivation to handle a task is an essential factor in how individuals cope with challenges [9].

Approaches that are active, interpersonal, and focused on solving problems are typically linked to increased self-esteem and positive emotions after a traumatic brain injury [9,10]. Poorer psychological outcomes, such as increased levels of depression and anxiety, have been associated with the use of strategies for escaping and avoiding situations following brain injury [10,11,12], as has the onset of post-traumatic stress disorder [13].

The purpose of this review is to analyze studies that explore how coping strategies influence psychological changes in patients with acquired brain injury. 

## 2. Materials and Methods

### 2.1. Search Strategy

A review was conducted on existing studies that examined how coping strategies can lead to psychological changes in individuals with acquired brain injury. The literature search was conducted via PubMed, Scopus, and Embase and included articles published from 2013 to 2023 using the following search keywords: ((((Traumatic Brain Injury [Title/Abstract]) OR (Acquired Brain Injury[Title/Abstract])) OR (Neurorehabilitation[Title/Abstract])) AND (Coping[Title/Abstract])) OR (Adaptive Skills[Title/Abstract]).

### 2.2. Inclusion Criteria

Studies that described or investigated coping strategies in patients with traumatic or acquired brain injury were included. Only articles written in English were included in the review.

### 2.3. Exclusion Criteria

Studies were excluded due to a lack of data or information on coping strategies in patients with acquired brain injury. Systematic, integrated, or narrative reviews were also excluded, although reference lists were examined and included if appropriate. Equally, all manuscripts written in languages other than English were excluded.

### 2.4. Study Selection

The procedure for selecting the studies involved three stages. Initially, articles not written in English and duplicates were eliminated. Next, we analyzed the titles and abstracts for relevance to the specific topic of coping strategies in patients with ABI. At the end, the full text of all potential articles was thoroughly analyzed to check the suitability of the study and the completeness of the data. We registered the review on OSF.io, and it is available at this link: https://doi.org/10.17605/OSF.IO/J8V6R (accessed on 15 May 2024).

## 3. Results

**Figure 1:**
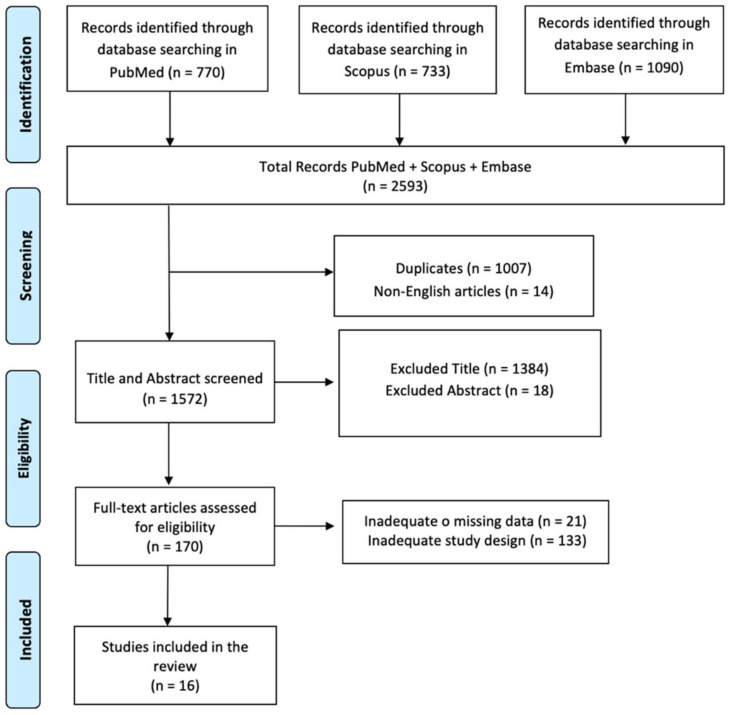
PRISMA flow chart for the current review.

The search produced 2593 articles as results. Subsequently, 1007 articles were removed because they represented duplicates, 14 articles were excluded because they were written in a language other than English, and 1402 articles were removed based on the title and abstract screening. In addition, 154 articles were removed based on the screening of inappropriate study designs and untraceable articles. Sixteen research articles met the inclusion criteria. The screening process is summarized in Figure 1, and the studies included in this review are shown in Table 1.

The articles included in this review studied coping and strategies in patients with traumatic or acquired brain injuries. The neurorehabilitation aspect was also considered as an integral part of the patients’ adaptive experience.

The different coping strategies and their influences on other psychological functions were evaluated in fourteen articles [14,15,16,17,18,19,20,21,22,23,24,25,26,27]. The impact of psychological intervention or training on coping and adaptive skills in ABI or TBI patients was assessed in two other articles [28,29].

### 3.1. Coping Strategies Can Improve Psychological Functions Post-ABI

#### 3.1.1. Association between Coping, Resilience, and Cognitive Function Post-ABI

Coping has been found to be associated with neuropsychological performance, level of education, and the amount of time elapsed since the brain injury. In one study, it was found that patients with high resilience exhibit lower levels of psychological distress due to their reduced tendency to make negative evaluations, such as perceiving threats or losses, and their tendency to use more active coping strategies compared to individuals with low resilience [14]. Another study proved that individuals who exhibited a more proactive coping style were less likely to report symptoms, while those who sought help more readily were more likely to report symptoms. There was no significant relationship between an avoidance coping style and the extent of the self-reported symptoms [15]. It was also demonstrated that individuals who self-reported executive dysfunction were more likely to use passive coping styles, which may have a detrimental effect on their quality of life and depressive symptoms after ABI. Furthermore, the results indicated that employing a problem-focused coping style was more beneficial for patients with fewer reported executive functioning difficulties, while it was less effective for those with greater difficulties in this area [16].

#### 3.1.2. Evolution of Coping Styles and Specific Strategies Post-ABI

One study analyzed the coping styles pre- and post- traumatic brain injury and found that: (i) pre-injury coping style was found to have a positive correlation with post-injury coping style; (ii) after the injury, an increase in nonproductive coping strategies and a decrease in productive coping strategies were linked to a poorer psychosocial outcome at the 1-year mark, and individuals who already had a high usage of nonproductive coping before the injury tended to experience worse outcomes; (iii) while productive coping did not consistently decrease over time, nonproductive coping showed a greater increase following the first year post-injury [17]. Patients with TBI who experience severe depressive symptoms tend to have higher levels of maladaptive personality traits and poorer coping styles, in contrast to those with mild, moderate, or no depressive symptoms, who exhibit both lower levels of maladaptive traits and more positive coping strategies [18]. One article proved that there are two strategies of coping for dealing with a TBI to consider in these patients: action/distraction and trivialization/resignation. The trivialization/resignation strategy was found to have a detrimental impact on various components of health-related quality of life, while the action/distraction strategy demonstrated positive and significant relationships with two domains. Additionally, both strategies displayed significant correlations with anxiety, depression, recovery, cognitive function, mood states, and the severity of trauma [19].

#### 3.1.3. Coping Mechanisms and Their Long-Term Effects Post-ABI

It was also found that after one year, individuals with ABI consistently utilized a narrow range of coping mechanisms in various circumstances. How distressing the specific challenges were did not significantly impact the coping strategies employed by the patients. The patients tended to adopt a more task-focused approach when facing cognitive difficulties as opposed to physical challenges. Those with greater confidence in managing symptoms related to their brain injury were less inclined to rely on emotion-based coping strategies and displayed less variability in their use of such strategies [20]. Another study has demonstrated the use of specific coping strategies during the chronic phase in patients with ABI. Avoidance coping styles appear to become more prevalent during the chronic phase after a brain injury over a six-month period, even for patients who receive psychoeducational and/or individualized treatment that does not specifically target changing coping styles [21]. One article verified a link between emotion-focused coping and a lower quality of life but found that individuals with high self-efficacy were able to counteract this negative impact. Boosting self-efficacy levels appeared to lessen the detrimental effects of emotion-focused coping on health-related quality of life, indicating that high self-efficacy serves as a protective factor [22].

#### 3.1.4. The Importance of Adaptive Coping and Self-Efficacy in Post-ABI Recovery

Ome study demonstrated that although adaptive coping is not correlated with distress, it plays a crucial role in fostering post-traumatic growth in patients with ABI, underscoring the intricate connections between coping, growth, and distress. Additionally, a strong sense of control over their ABI treatment is significantly linked to the utilization of adaptive coping mechanisms [23]. Patients with a mild traumatic brain injury also maintain two specific coping styles over time after 1 year: the passive coping type and emotion expression. One study discovered intriguing connections between coping strategies and self-efficacy, showing that individuals with high levels of self-efficacy tended to use an active coping style, while those with lower self-efficacy gravitated towards a passive/avoidant approach [24]. Changes over time were observed in the utilization of various coping strategies, such as active coping, distraction seeking, avoidance, seeking social support, and positive reframing. Overall, there was a decrease in the frequency of these coping methods from the two-week mark to twelve months, with most strategies showing a consistent decline [24]. The only exception to this trend was positive reframing, which remained relatively stable over time [24].

#### 3.1.5. Adaptive Coping and Depression

Another article demonstrated that patients with TBI who utilize adaptive coping strategies are likely to lessen the impact of information processing speed on self-reported depression [25]. A significant correlation was found between the use of negative or avoidance-based coping methods and increased levels of affective symptoms in patients with acquired brain injury [25]. Approach-based coping techniques were more commonly used by ABI patients without significant clinical issues [26]. Among highly symptomatic ABI patients, emotional discharge was the most frequently employed avoidance strategy, indicating a tendency to release tension through the expression of negative emotions. These patients also showed a significant use of cognitive avoidance, which involves unrealistic conceptualization of stress or problems, as well as acceptance/resignation, where stress is accepted without significant efforts to address the situation [26].

#### 3.1.6. Coping Strategies in Adolescents Post-ABI

One last study with adolescent with TBI demonstrated that these patients employ accommodative coping strategies, such as engaging in downward comparisons (such as “it could be worse”), accepting unresolvable issues, reframing their perspective on the problem in a more positive light, shifting their attention to new interests, and prioritizing rest and balance [27]. The coping strategies employed by patients with ABI and TBI depend on factors such as the severity of the diagnosis, their age, the duration of living with the condition, and individual characteristics, all of which impact their quality of life and rehabilitation outcomes [27].

### 3.2. Psychological Rehabilitation and Coping Strategies in ABI Patients

Confirming the study’s premise, it was demonstrated that a 5-week rehabilitation intervention program incorporating cognitive behavioral therapy and mindfulness-based stress reduction resulted in a significant improvement in calming and distractive coping strategies among prisoners who had suffered traumatic brain injuries. The intervention, which included psychoeducation on brain injuries, showed moderate effectiveness in the intervention group. However, the improvement in coping strategies did not persist up until the 12-week follow-up assessment [28].

Coping strategies can be learned and then taught. In one research work, the use of virtual reality cognitive rehabilitation resulted in enhanced executive function and coping strategies according to targeted timed exercises, leading to an overall cognitive improvement. The VRRS group not only showed significant enhancements in their cognitive functions but also demonstrated an improvement in their coping strategies and problem-solving abilities, indicating a correlation with divided attention tasks [29]. In addition, improvements in depressive symptoms were found according to the Hamilton Rating Scale for Depression (HRS-D). Reducing depressive symptoms not only improves emotional well-being but can also have a positive impact on other cognitive functions and coping skills, creating an overall improvement in quality of life. These results highlight the potential of the VRRS as an effective tool for cognitive rehabilitation and mental well-being, offering an innovative and engaging solution to cognitive and emotional challenges [29]. Patients with TBI may find it advantageous to undergo a personalized rehabilitation program that focuses on implementing active and effective coping strategies [16].

## 4. Discussion

Our review aimed to examine the role of coping strategies in psychological changes in patients with acquired brain injuries. All the studies included in this review have demonstrated the coping strategies influence ABI patients’ ability to face or avoid their clinical and emotional experience with the disease. Indeed, psychological and clinical variables such as resilience, self-efficacy, problem orientation, proactivity/passivity, the type and degree of acquired brain injury, social/family support, and prospects of quality of life with the disease all influence a patient’s experience and their rehabilitation process, even after years [14,15,16,17,18,19,20,21,22,23,24]. Research has shown that patients with ABI who use active coping strategies and are problem-oriented face the disease with greater resilience and a sense of self-efficacy and report fewer symptoms [14,18,20].

Instead, patients who use passive coping strategies and avoid the situation or their emotions through trivialization or resignation are individuals predisposed to depression and a worse quality of life. Strategies based on avoidance predispose this type of patient to releasing tension, derived from a cognitive evaluation and awareness of their diagnosis, through the expression of negative emotions, showing higher symptoms [15,18,19,24,25,26]. From a rehabilitation point of view, studies report the possibility of being able to teach patients with ABI functional coping strategies based on dealing with stressful situations and on proactivity through cognitive behavioral techniques, stress reduction treatments based on awareness, and psychoeducation about traumatic brain injury [28]. The use of modern technologies, such as a virtual reality rehabilitation system, has proven effective in enhancing and training the cognitive functions of patients with ABI and predisposing them to problem-oriented coping strategies, together with having positive effects on general mood [29].

Based on the evidence gathered in this review, we can affirm that coping mechanisms act as a reservoir of emotional resilience within an individual and influence how they respond to perceived stress, whether it originates from within themselves or externally. These mechanisms can either aid in effectively managing the immediate situation or have negative consequences on a person’s psychological well-being in the long run. The scientific literature highlights that human beings dealing with a stressful situation use certain strategies or psychological mechanisms to cope with it. Specifically, two main strategies exist for these individuals: (i) problem-focused coping strategies, used by those who tend to address the underlying causes of their problems, trying to understand them and learning new skills that can allow them to better manage them with the aim of eliminating the source of a stressful event, and (ii) emotion-focused coping strategies, in those who try to manage the emotions that arise during a stressful or conflictual event in an attempt to alleviate, reduce, or prevent the discomfort resulting from them. Another criterion for the classification of coping skills, similar to the previous criterion but which differs due to the non-centrality of the object with which one intervenes, is the distinction between coping strategies aimed at avoidance, therefore distracting oneself and not thinking about the problem, and strategies aimed at an approach, that is, not only facing the threat but also being vigilant about information related to it [30]. According to the current research, some stable personality characteristics are associated with the adoption of certain coping styles thanks to their influence on an individual’s cognitive evaluation of events [31].

Dealing with ABI pathologies is often viewed as a stressful situation that presents many challenges. As a result, individuals may struggle to maintain control over their lives. Coping resources play a crucial role in preserving feelings of self-worth and managing the demands of the disease. The presence of coping resources can impact how individuals perceive their situation and empower them to handle their illnesses effectively [32]. Studies have shown that psychological coping resources, such as mastery, self-esteem, and self-efficacy, can have a positive impact on how individuals cope with declining health [33,34,35,36]. It is believed that the establishment of effective coping mechanisms is integral to a growth journey, crucial for healthy social–emotional development, and essential for overall well-being [37,38]. Indeed, coping techniques that are described as active, interpersonal, and focused on solving problems have been linked to increased self-esteem and positive emotions after a traumatic brain injury [39]. After suffering a traumatic brain injury, individuals may face ongoing physical, vestibular, cognitive, and emotional challenges that can significantly affect their daily life, ability to make decisions, and relationships with others [40,41]. Mindfulness-based stress reduction therapies have been shown to be effective in individuals who exhibit aggressive behavior issues [42]. Knowing the factors that can impact coping styles can help tailor rehabilitation efforts to individuals who may struggle to adapt on their own. Some research suggests that active problem-solving coping styles rely on cognitive abilities, so cognitive impairments post-brain injury could hinder the use of this approach, leading to a greater reliance on emotion-focused coping mechanisms [43,44,45,46,47]. The structured approach of cognitive behavioral therapy is considered beneficial for individuals with traumatic brain injury [47]. Enhancing coping strategies following TBI could potentially boost both executive function and mood, as there are connections between these factors [48].

Based on the research identified, we can confirm that various aspects determine the utilization of coping strategies in patients with ABI: cognitive (the assessment that is developed regarding one’s clinical condition with the prospects of recovery and rehabilitation); emotional (awareness of one’s health and psychological state); and personality traits (characteristics or predispositions to tending towards one response mode over another). Psychological variables, such as resilience, sense of self-efficacy, and predisposition to anxiety or depression, and the type of cognitive assessment carried out for patients with ABI can provide us with various information on the possible coping strategies that they will potentially use. Conversely, an assessment of how an individual deals with the situation after receiving a diagnosis of a medical condition can provide clinicians with information on their emotional response methods and personality traits. Both clinical reasoning paths lead to the same goal: obtaining a more specific clinical and psychological picture of the cognitive, emotional, and behavioral response methods.

### 4.1. Strengths and Limitations

The main strength of this review article is its analysis of coping strategies that can aid clinical recovery and mitigate stressful events after brain injury. Indeed, coping strategies produce psychological changes in patients and influence the way they cope with their clinical condition for individuals with traumatic or acquired injuries. This aspect highlights another important feature: the importance of evaluating these strategies both before and after the onset of the disease. Knowing the patient’s pre-morbid and current strategies will in fact make it possible to understand whether there have been changes and possibly allow rehabilitation and/or empowerment processes to be modulated based on the patient’s current abilities.

However, the present study also has limitations. First of all, although the purpose of the review was not to systemically examine all approaches, not including some databases may have left out some significant results. Furthermore, the heterogeneity of the studies with respect to the sample size, scales used, and outcomes measured made it impossible to make any conclusive statements regarding the effectiveness of the interventions proposed by the individual studies.

### 4.2. Future Perspectives

Planning and preparing rehabilitation plans built specifically for these patients that consider the possibility of them learning new coping strategies can mitigate the negative effects of the illness and enhance their quality of life. A recovery-oriented approach allows people to use their strengths and the resources in their living environment. Having more control over your life and managing your mental health are among the key factors in regaining self-respect and self-esteem [49]. Within a hospital context, the true objective of treatment must be “recovery” [50], understood as the best possible level of personal and social functioning and integration into one’s environment, and this necessarily requires interpersonal and social skills. A person without coping skills is much more likely to fail in various domains of functioning and, as a result, experience anxiety and frustration, higher levels of stress, and a greater risk of relapse. Therefore, teaching these patients new strategies through specific training would reduce the impact of their persistent symptoms while at the same time reducing the high levels of discomfort and psychological distress associated with this clinical condition.

Although the characteristics of hospital and ward environments differ from sheltered residential or home settings, it is possible to teach effective coping strategies through training by expert psychologists and clinicians.

Future research could investigate how learning coping strategies in patients is influenced by and adapted to contextual variables.

## 5. Conclusions

This review demonstrated that coping strategies have an impact on different aspects of the clinical and personal lives of patients with ABI. The rehabilitation process must consider the influence of these mechanisms on dealing with situations, as they can change the cognitive and emotional perceptions of experience with the disease, as well as laying the foundations for function or dysfunction in terms of the propensity of a person for the path of psychological and physical recovery. Subsequent research on how to learn functional and positive coping strategies to face this disease or accept one’s awareness of one’s diagnosis for life could open up new clinical perspectives.

## Figures and Tables

**Table 1:** Summary of studies included in the research.

Author	Aim	Study Design/ Intervention	Treatment Period	Sample Size	Outcomes Measures	Main Findings
Scholten et al. 2020 [14]	To explore how personal resource resilience is related to psychological distress and whether perceptions of threat and loss, as well as passive coping strategies, play a mediating role in this connection.	A cross-sectional study	Not specified	228 people	Hospital Anxiety and Depression Scale; Connor–Davidson Resilience Scale-10; Appraisals of Life Events scale; Utrecht Coping List	This study findings suggest that the connection between resilience and psychological distress is influenced by appraisals of threat and loss, as well as passive coping strategies.
Anderson et al. 2018 [15]	To examine the potential connections between coping style and/or illness perceptions and the intensity of self-reported symptoms of post-concussion syndrome (PCS) following a mild traumatic brain injury (mTBI) during the post-acute phase.	Prospective study	6–12 weeks	61 adults (44 male, 17 female) aged 18–60 years	Rivermead Post-Concussion Symptoms Questionnaire (RPQ)	This study suggests that early and late enduring types of PCS symptoms are linked to distinct coping strategies and perceptions of illness, which could impact the way self-reported symptoms are managed and treated following an injury.
Gregorio et al. 2015 [16]	To examine the interconnections between executive functioning, coping mechanisms, depressive symptoms, and quality of life in individuals who experience neuropsychiatric symptoms after ABI.	Cross-sectional study	Not specified	151 patients	Stroop Color Word Test, Trail Making Test, Frontal Systems Behavioral Scale, Patient Health Questionnaire, Utrecht Coping List, and Life Satisfaction Questionnaire	The authors found that people who struggle with executive functioning after an ABI are more likely to rely on ineffective passive coping mechanisms, which need to be addressed during treatment. It is not advisable to encourage individuals facing executive functioning challenges to engage in problem-focused coping strategies.
Gregorio et al. 2014 [17]	To investigate how self-reported coping strategies before a TBI impact individuals’ coping, psychosocial and emotional well-being, and overall quality of life one year after the injury.	Prospective, longitudinal design	36 months	One hundred seventy-four participants with TBI	Quality of Life Inventory; Coping Scale for Adults–Short Version; Hospital Anxiety and Depression Scale; Sydney Psychosocial Reintegration Scale	The results suggest that identifying individuals at risk of using unhelpful coping strategies and experiencing negative psychosocial outcomes after TBI is important. Furthermore, the findings highlight the importance of promptly implementing interventions to promote helpful coping behaviors and decrease reliance on unhelpful coping mechanisms to achieve positive long-term psychosocial outcomes.
McIntyre et al. 2020 [18]	To investigate the relationship between coping strategies, personality traits, and depression symptoms in individuals with ABI.	Observational cohort study	2 weeks	89 individuals	Acceptance and Action Questionnaire; Patient Health Questionnaire, 9-item (PHQ-9); Anxiety Sensitivity Index; Adult Dispositional Hope Scale; Rosenberg Self-Esteem Scale; Big Five Inventory; and Brief Coping Orientation of Problems Experienced	The authors found that the adoption of passive coping mechanisms along with heightened emotional avoidance behaviors in severely depressed individuals with ABI may result in adverse long-term consequences. Interventions focused on promoting active coping strategies and decreasing emotional avoidance tendencies could be advantageous in mitigating the disability and diminished quality of life linked with depression in individuals with ABI.
Sasse et al. 2014 [19]	To examine how individuals cope with TBI and the impact of their coping strategies on health-related quality of life. (HRQoL).	A follow-up study using a cross-sectional design	Not specified	141 adults	Short Form-36 Health Survey (SF-36); Freiburg Questionnaire of Coping with Illness (FQCI); HRQoL by the Quality of Life after Brain Injury (QOLIBRI) scale.	This study showed that two coping factors were pinpointed, showing varying effects on HRQoL. The use of maladaptive coping strategies was found to significantly impact HRQoL, with lower reliance on these strategies correlating with better HRQoL outcomes.
Brands et al. 2014 [20]	To examine how patients with newly acquired brain injuries adapt to challenges, focusing on the impact of the problem type, self-efficacy, self-awareness, and self-reported executive functions on their coping abilities.	A prospective study	1 year	136 patients	Coping Inventory for Stressful Situations (CISS)	This study found that individuals who have suffered from acquired brain injuries often depend on a specific set of coping strategies to manage various situations over time. Having a strong belief in their ability to handle challenges increases their likelihood of using proactive coping strategies. However, difficulties with executive functioning may hinder their ability to choose effective coping strategies.
Gregorio et al. 2016 [21]	To investigate the factors that influence coping mechanisms in patients during the chronic phase following an acquired brain injury characterized by significant neuropsychiatric symptoms.	Comparative study	The duration of the treatment was different for each individual and determined by the time that the patients needed to meet their own goals.	A total of 42 patients and 32 significant others participated.	Neuropsychiatric Inventory (NPI), the AQ, the FrSBe at home or at the center, Trail Making Test, and the Stroop Color Word Test (Stroop). questionnaires included the Utrecht Coping List (UCL), Frontal Systems Behavioral Scale (FrSBe), the Patient Health Questionnaire (PHQ-9), Awareness Questionnaire (AQ), and the Life Satisfaction Questionnaire (LiSat-9).	This study showed that avoidance coping increased during the chronic phase following a brain injury. Changes in coping strategies were found to be linked to the severity of the neuropsychiatric symptoms and self-awareness levels rather than self-reported executive functioning. This highlights the importance of incorporating these factors into treatment programs.
Brands et al. 2014 [22]	To explore how self-efficacy and coping strategies are related to quality of life (QOL) and social participation and to examine the impact of self-efficacy levels, changes in self-efficacy, and coping styles on long-term QOL and social participation.	Prospective clinical cohort study	2 to 4 weeks	148 patients with a newly acquired brain injury	Frenchay Activities Index, Coping Inventory for Stressful Situations, EuroQuol 5D (the EQ-5D index and the EQ-5D visual analog scale [EQ VAS]), Traumatic Brain Injury Self-Efficacy Questionnaire, and the 9-item Life Satisfaction Questionnaire (LiSat-9)	The results indicated that self-efficacy and coping strategies are strong predictors of long-term quality of life, although they may not have as significant of an impact on long-term social participation. Having a high level of self-efficacy can help counteract the negative effects of emotion-oriented coping. Improving self-efficacy in the early stages following an ABI could lead to positive long-term outcomes.
Rogan et al. 2013 [23]	To explore how illness perceptions, distress, disability, coping strategies, and overall health contribute to post-traumatic growth in individuals with ABI.	Cross-sectional study	Not specified	70 people with an ABI	Post-Traumatic Growth Inventory, Functional Independence Measure and Functional Assessment Measure, Brief-COPE, Hospital Anxiety and Depression Scale, Illness Perception Questionnaire Revised	This research further supports the factors linked to post-traumatic growth, specifically focusing on how adaptive coping strategies could aid in promoting growth. However, more research is needed to determine the exact nature of this relationship.
Scheenen et al. 2017 [24]	To examine the consistency of various coping styles over a one-year period post mTBI and explore the connection between coping styles and levels of self-efficacy.	A prospective cohort study	January 2013 to December 2015	425 mTBI patients	The Utrecht Coping List (UCL)	This study showed that the majority of coping styles experienced a decline over time, with the exception of positive reframing, which initially decreased before rebounding. It is worth noting that the passive coping style remained relatively stable within the first year post-injury.
Spitz et al. 2013 [25]	To investigate the relationships between cognition, coping strategies, and emotional adjustment after TBI, considering both direct and indirect effects, as well as moderating factors.	Cross-sectional single-group design	19 months	97 participants with mild to severe TBIs	Coping Scale for Adults; BIRT Memory and Information Processing Battery; Doors Test from the Doors and People Test; Digit Span Symbol Digit Modalities Test—Oral Version; Hayling Sentence Completion Test; Trail Making Test; Hospital Anxiety and Depression Scale; Controlled Oral Word Association Test.	This study found that individuals who exhibited lower performance on memory, executive functions, attention, and information processing tasks also reported higher levels of depression and anxiety. There was no direct relationship found between cognitive abilities and emotional adjustment. However, the application of adaptive coping mechanisms was shown to influence the connection between information processing speed and self-reported depression.
Velikonja et al. 2013 [26]	To assess how a combination of emotional profiles from both Axis I and II, along with demographic and psychosocial factors, influences coping responses in individuals with TBI.	Retrospective study	Not specified	100 patients	Coping Response Inventory (CRI) and Personality Assessment Inventory (PAI)	The results suggest the coping mechanisms utilized by individuals with brain injuries are influenced by symptoms from Axis I and II disorders, along with factors such as psychosocial support, stress levels, relationships, and gender.
Mulligan et al. 2023 [27]	To examine past qualitative descriptions provided by young adults who experienced a TBI during adolescence, with the goal of investigating how they coped and recovered during this crucial developmental period.	A qualitative interview-based study	Not specified	Thirteen adults	Semi-structured individual interviews	This study showed that maximizing recovery from a TBI sustained during adolescence could be achieved by improving our understanding of the unique impacts on young individuals among both clinicians and families; consistently monitoring symptoms over the long term, including emotional responses; and providing ongoing emotional support.
Mitchell et al. 2021 [28]	To assess the efficacy of a psychological intervention in enhancing coping mechanisms, reducing post-concussion symptoms, and minimizing instances of rule violations in incarcerated adult males who have a prior traumatic brain injury.	A single-center randomized, wait-list pilot study	Not specified	55 adult male participants	The Negative Affect Repair Questionnaire and Rivermead Post-Concussion Symptom Questionnaire were completed at baseline, post-intervention (5 weeks), and at 12-week follow-up.	This study showed that manualized psychological intervention has the potential to help prisoners with a history of traumatic brain injury develop positive coping strategies.
De Luca et al. 2023 [29]	To explore the impact of non-immersive virtual reality training on enhancing executive functions and alleviating symptoms of anxiety and depression in individuals with TBI.	A pilot study	24 one-hour sessions (3 times a week for 8 weeks)	20 patients with moderate to severe TBIs	Coping Orientation to the Problems Experiences—new Italian version (COPE-NIV). Trail Making Test (TMT). Montreal Cognitive Assessment (MoCA). Hamilton Rating Scale for Depression (HRS-D). Frontal Assessment Battery (FAB).	The results suggest that the use of virtual reality (VR) rehabilitation with the Virtual Reality Rehabilitation System (VRRS) could be an effective and engaging method to enhance visuo-executive skills, coping mechanisms, and mood in individuals with chronic TBI.
